# Total Hip Replacement in Developmental Hip Dysplasia: A Narrative Review

**DOI:** 10.7759/cureus.14763

**Published:** 2021-04-29

**Authors:** George C Papachristou, Eleni Pappa, Dimitrios Chytas, Panagiotis T Masouros, Vasileios S Nikolaou

**Affiliations:** 1 2nd Department of Orthopaedics, School of Medicine. National and Kapodistrian University of Athens, Athens, GRC; 2 5th Department of Orthopaedics, "KAT" General Hospital of Athens, Athens, GRC; 3 Department of Orthopaedics, European University of Cyprus, Nicosia, CYP; 4 Department of Orthopaedics, General Hospital Evangelismos, Athens, GRC; 5 2nd Department of Orthopaedics, National and Kapodistrian University of Athens School of Medicine, Athens, GRC

**Keywords:** developmental hip disease, total hip replacement, hip arthritis, total hip arthroplasty, congenital hip dysplasia

## Abstract

The reconstruction of the hip joint in patients suffering from developmental hip dysplasia (DDH) is a demanding procedure and presents many challenges to the reconstructive surgeon. Higher rates of mechanical complications are present in this group of patients. The results of cemented and uncemented implants used in DDH patients are very promising, according to recent outcomes. However, the surgeon has to be aware of several complications, in order to establish an uneventful surgical management of DDH. The specific article investigates the technical challenges and clinical results of total hip arthroplasty in patients with DDH.

## Introduction and background

**Figure 1:**
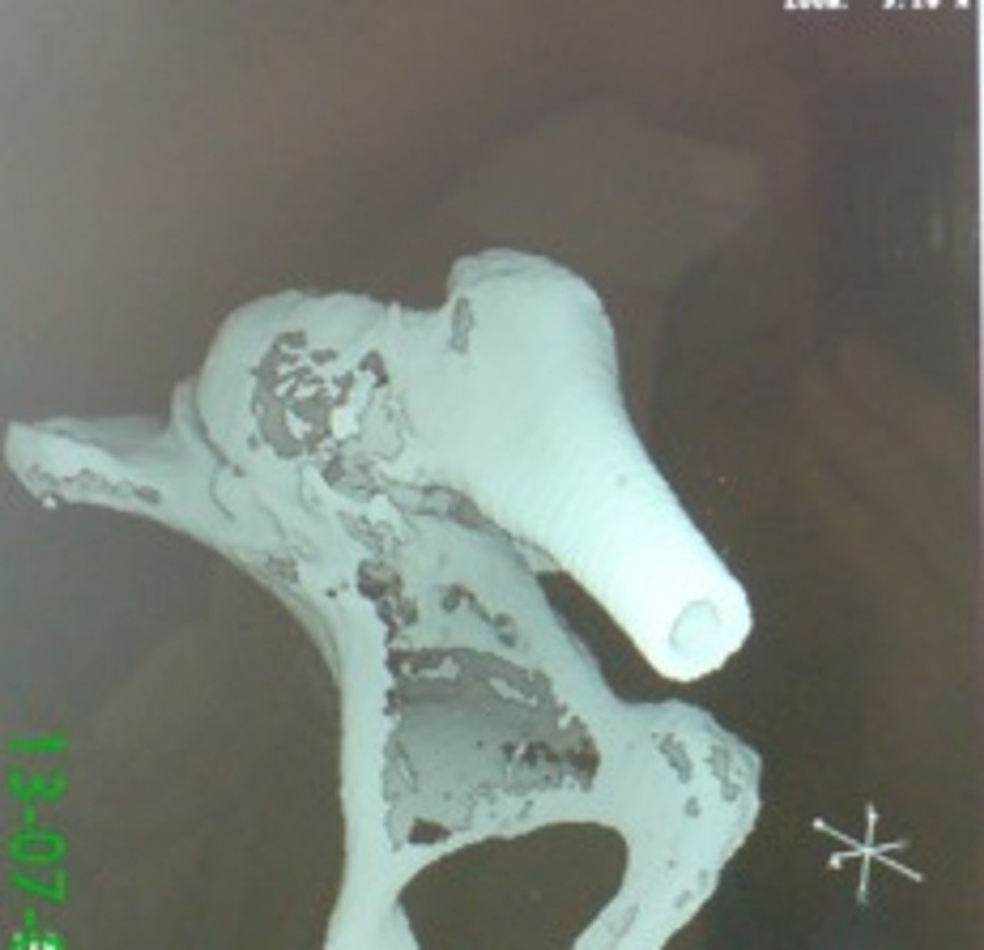
3D hemipelvis remodelling from CT scan of dysplastic hip.

Developmental hip dysplasia (DDH) encompasses a spectrum of progressive changes to both the femur and the acetabulum, which eventually lead to secondary osteoarthritis of the hip joint. The abnormal bone morphology, growth and orientation, as well as soft tissue alterations, completely distort the normal relation between acetabulum and femoral head. Under these circumstances, THA in this population can be unpredictably challenging [1] with a reasonably high complication rate, compared to primary osteoarthritis. Aside from the morphological challenges, surgeons must also consider the usually younger age of the patients and the subsequent need for revision surgery [2]. An understanding of the common acetabular and femoral morphologic abnormalities will aid the surgeon in preparing for the complexity of the surgical case. In this article, we attempt a comprehensive review of the technical challenges and outcomes of THA in patients with DDH, including emphasis on the most suitable acetabular and femoral components and the potential reasons for postoperative complications and their management. A systematic search of the PubMed database was conducted using “dysplastic hip” and “total hip arthroplasty” as key phrases from 2000 to 2020. Studies involving pediatric patients were excluded (Figure 1). 

## Review

Classification

The significant heterogeneity of dysplastic hips necessitates a classification system, so that investigators could use the same terminology and compare their results. One of the most widely used classifications, as proposed by Crowe et al. [3], is based on the extent of proximal displacement of the femoral head with respect to the vertical height of the pelvis. Alternatively, proximal migration can be expressed by dividing the distance between the inter-teardrop line and the femoral head-neck junction with the line connecting the ischial tuberosities and the line connecting the iliac crests, being characterized in the current literature as quantitative. Hartofilakidis et al. classification, which ensued, describes three distinct entities: mildly dysplastic, low and high dislocation, based on the anatomical abnormalities. In the case of the aforementioned classification, it has been mentioned in the current literature that it is lacking in providing information regarding the anatomical structural changes especially during hip surgery [4-6]. However, both classifications have been used for both clinical practice and research purposes. 

Both classifications were evaluated as having substantial to almost perfect inter- and intraobserver agreement [7]. Kose et al. [8] found substantial inter- and intra-observer agreement for Crowe classification and substantial to moderate agreement for the Hartofilakidis one. We agree with Kose et al. [8], who concluded that each system has its own advantages and disadvantages, suggesting the need for both of these classifications together to increase accuracy. Nevertheless, we consider that Hartofilakidis et al classification is simpler than the one by Crowe et al., as it describes better the anatomical differences that the surgeon faces during the operation. 

Recently Gaston et al. [9] described a new classification system for adult DDH with separate classifications for acetabulum and femur, whereas Clavé et al. [10,11] underlined the significance of the preoperative planning of total hip replacement in DDH by using both classification systems (Tables 1, 2, 3).

**Table 1:** Classification system of congenital hip disease in adults according to Crowe et al.

Type I	<50% femoral head subluxation
Type II	50%-75% femoral head subluxation
Type III	75%-100% femoral head subluxation
Type IV	>100% femoral head subluxation

**Table 2:** Classification system of congenital hip disease in adults according to Hartofilakidis et al.

Dysplastic hip	The femoral head is contained within the original acetabulum despite the degree of subluxation.
Low dislocation	The femoral head articulates with a false acetabulum that partially covers the true acetabulum.
High dislocation	The femoral head is completely out of the true acetabulum and has migrated superiorly and posteriorly.

**Table 3:** Classification system of congenital hip disease in adults according to Gaston et al.

AI	Dysplastic acetabulum
AII	The acetabulum associated with a low femoral dislocation
AIIIa	The post-surgical acetabulum: with retained metalwork
AIIIb	The post-surgical acetabulum: without retained metalwork

Surgical pearls and challenges

Elaborate preoperative planning and availability of a wide range of implants are imperative. The morphological changes, the location of the native acetabulum, the extent of the proximal displacement of the femoral head and the combined anteversion all need to be considered pre-operatively. Standing full-length X-rays are used to assess leg-length discrepancy (LLD) and determination of the subtrochanteric osteotomy. The accurate size of the prosthesis as well as the specific site of the acetabular cup position should also be determined. Cups and heads of a smaller size need to be available on site. 3D preoperative planning can be a useful asset. Current literature suggests that CT-based three-dimensional templating provides the best accuracy in predicting the anatomic location of the acetabulum, but also the acetabular bone stock and bony landmarks. Fluoroscopy has also been used but the 2D images are considered to overestimate the present bone adequacy due to the bone shield.

In mildly dysplastic hips, standard approaches including anterior, anterolateral, and posterior can be utilized depending on surgeon’s preferences. However, in cases of severe dysplasia a posterior approach is favored to allow for adequate exposure of the femoral head and acetabulum [12].

Reconstruction of the acetabular side is the most problematic part due to the deficient bone stock. Identification of the true acetabulum and re-establishment of the normal hip center are crucial, although not always feasible [13]. After resection of the femoral head, three steps can aid in locating the native acetabulum: (a) the ligamentum teres is followed distally to the cotyloid fossa, (b) the inferior capsule is identified, which is likely to be in line with the transverse ligament, (c) the bony landmarks (anterior and posterior). A triangle-shaped fossa, inferior to the pseudoacetabulum, which typically contains fatty tissue reveals the location of the true acetabulum. The native acetabulum has usually a small diameter requiring smaller components (usually 38 to 52 mm), in comparison with primary osteoarthritis [14]. If the pseudoacetabulum is mistakenly identified as the native one, this can result in an oversized acetabular cup, placed in a high and lateral position [13].

Medialization of the anatomic hip joint, in order to accommodate the whole cup, can be achieved by means of either controlled reaming and deepening of the acetabulum or the so-called cotyloplasty (osteotomy of the medial acetabular wall and medial advancement) [15,16]. However, cotyloplasty has been associated with complications such as cup migration, loosening and osteolysis. Failure of the initial fixation and protrusion of the cup into the pelvis have also been reported. Moreover, revision after cotyloplasty, can be troublesome due to the thin medial wall.

The femur in DDH is generally characterized by increased neck-shaft angle, excessive anteversion and a relatively small diaphyseal diameter [13]. Interestingly, Sugano et al. [17] reported an anteversion of more than 40 degrees in 25% of their patients, while Noble et al. [18] also calculated a mean increase in femoral anteversion by 5 to 16 degrees. The anteversion can be adjusted precisely using either a modular stem or the modified S-ROM stem which is based on 3D reconstruction of proximal femur based on CT data. Regarding monoblock stems, the findings in the literature are debatable. On the one hand, these stems may not be suitable for severe DDH, on the grounds that stem anteversion after THA with a tapered wedge can be greater in comparison with a metaphyseal filling stem [19-21]. On the other hand, in the case of severe femoral distortions where no merchandise implants may be available, a customized femoral prosthesis is likely to be used based on the principles of computer-aided engineering. 

Besides bony changes, soft tissue alterations should also be taken into account. The articular capsule is thickened and upwards elongated, while the function of the abductors is impaired. With the possible exception of the abductors, all the muscles around the hip joint are shortened in both low and high dislocations [22,23]. In most cases, brave intraoperative soft tissue release and tenotomies are advocated [24,25,26,27]. Wu et al. [28] described a soft-tissue release in four steps, starting with splitting of the adductor and parts of the iliotibial tract, followed by release of the iliopsoas tendon from the lesser trochanter, release of the attachments of the piriformis and hamstring muscles and finally osteotomy and advancement of the greater trochanter distally along with the gluteus medius insertion. Finally, the sciatic nerve is shortened, while the courses of the femoral nerve and the profunda femoris artery are distorted, thus putting them at increased risk during surgery.

Outcomes

Current evidence suggests that THA improves both the pain and the functional hip scores in patients with DDH. Inao and Matsuno [29] reported satisfactory mobility and improvement of pain after a mean follow-up of 12.9 years. Despite the overall functional improvement, patients suffering from severe DDH may continue to limp even post-operatively due to abductors’ weakness.

Cemented acetabular reconstruction is falling out of favor due to the decreased survival rates. Despite the fact that cemented cups were combined with autogenous bone graft, the revision rate was satisfactory. However, these rates worsen dramatically due to graft collapse or socket loosening [11,14]. Uncemented acetabular components, with or without bone augmentation, are now widely used in mildly dysplastic hips, with considerably low revision rates in mid- and long-term follow-up. A Kaplan-Meier survivorship analysis predicted a rate of survival of the acetabular component of 96% at 15 years. 

Concerning the use of the high hip center, the reports are limited in size. Perka et al. [26] stated that very good results were achieved in a relatively young patient population when the hip joint center had been properly restored, even if a small cup with a thin polyethylene liner was used. It is emphasized that the problems encountered during restoration of the center of rotation in THA for DDH correlated well with the severity of the dislocation and the clinical outcome was not found to be related with the severity of dislocation [30-33].

On the femoral side, the use of cement stems yield better outcomes compared to the acetabular side. However, the results are still inconsistent. In case of uncemented femoral components, the long-term results in DDH are lacking. The use of proximally fit uncemented components in hip dysplasia is challenging due to significant deformity, and often osteotomies and modular components are necessary to achieve an optimal fit [33]. Recently, Zhen et al [34] evaluated the results of uncemented press-fit Wagner femoral stems in patients with DDH in a mean follow-up of 7.7 years, where Harris hip score was improved, without any femoral fractures or stem revisions.

Femoral shortening via proximal femoral osteotomy and greater trochanter distal advancement can be associated with significant complications, while they allow for preservation of abductor mechanism as well as more flexibility in correction of rotational deformities. Patients with Crowe Ⅳ dysplastic hips treated with subtrochanteric osteotomy and uncemented components show generally excellent healing rates of the osteotomy, while a 10-year follow-up study also revealed improved lasting Harris hip scores in patients with dysplasia treated with modular femoral components and subtrochanteric osteotomy [34,35]. 

THA after previous joint preserving procedures, such as Salter, Chiari as well as the rotational acetabular osteotomy (RAO) or femoral osteotomies is considered as particularly demanding. However, Fukuri et al. reported similar functional results to patients who underwent THA in the presence of a rotational acetabular osteotomy [21]. Even in the case of the Bernese osteotomy, Parvizi et al. concluded that subsequent THA for DDH was not compromised [22] . On the other hand, poorer functional outcomes have been reported after combined periacetabular osteotomy (PO) and intertrochanteric valgus osteotomy [4]. On the femoral side, pre-existing osteotomies are associated with increased complications rate such as intraoperative fracture and nerve palsies. Though, the survivorship of THA after femoral osteotomies ranges from 43.7% to 100% [22].

Complications

The complications rate after THA in DDH patients is substantially higher when compared to THA due to primary osteoarthritis. They include fractures, dislocations, non-unions, loosening of the implants, neurovascular injuries, infections and impaired functional outcome. 

Such complications come from the hypoplastic bones (deficient acetabulum and upper femur) and the anatomical alterations. Moreover, there are difficulties due to the hypoplastic bones and the necessity to insert especially designed implants for DDH cases. 

Regarding sciatic nerve palsy, it is generally accepted that the extent of the lengthening should not exceed 3-4 cm. When osteotomy is applied intra-operatively, the sciatic nerve injury level ranges from 5% to 11.3%. Eggli et al. [25] proposed that the main reason of the sciatic nerve injury is direct intra-operative damage rather than leg length discrepancy. Care should be taken intra-operatively by applying controlled tension and femur traction during reduction. Intra-op nerve monitoring has been also proposed for high riding DDH [36]. 

Intraoperative fractures are likely to occur in 5.2%-26.8% of THA in cases of DDH [37-40]. Otherwise, if the femoral canal is too narrow to allow the insertion of the smallest stem, splitting of the femur, as proposed by Li et al. [27], is an alternative. This is mainly due to the narrow femoral canal and can be successfully treated intra-operatively with wire-plate internal fixation [41,42]. 

LLD is another common complication. Gait asymmetry is likely to occur when LLD > 10 mm exists. Lumbar scoliosis, pelvic tilt and severe limp should be taken into consideration to prevent under-correction of limb length. In cases of severe leg length discrepancy, a subtrochanteric osteotomy can be performed [12]. Several techniques, including transverse, oblique, z-shaped, or the double chevron osteotomy, have been proposed [23,24]. Current evidence suggests that THA combined with sub-trochanteric osteotomy is likely to provide satisfactory functional results [22,37,38,39]. If clinically significant limping is mentioned post-operatively, physical therapy and a shoe lift should be considered, as well as re-operation. In cases of bilateral DDH, LLD should be addressed by THA of the contralateral hip .In cases of irreducible dysplastic hips with expected lengthening greater than 3 cm , a two-stage procedure can be considered. In a case series of 17 patients with Crowe III and IV DDH, Yoon et al. concluded that two-stage THA using initial skeletal traction after extensive soft-tissue release, could provide an acceptable alternative solution with satisfying functional results [43]. 

**Figure 2:**
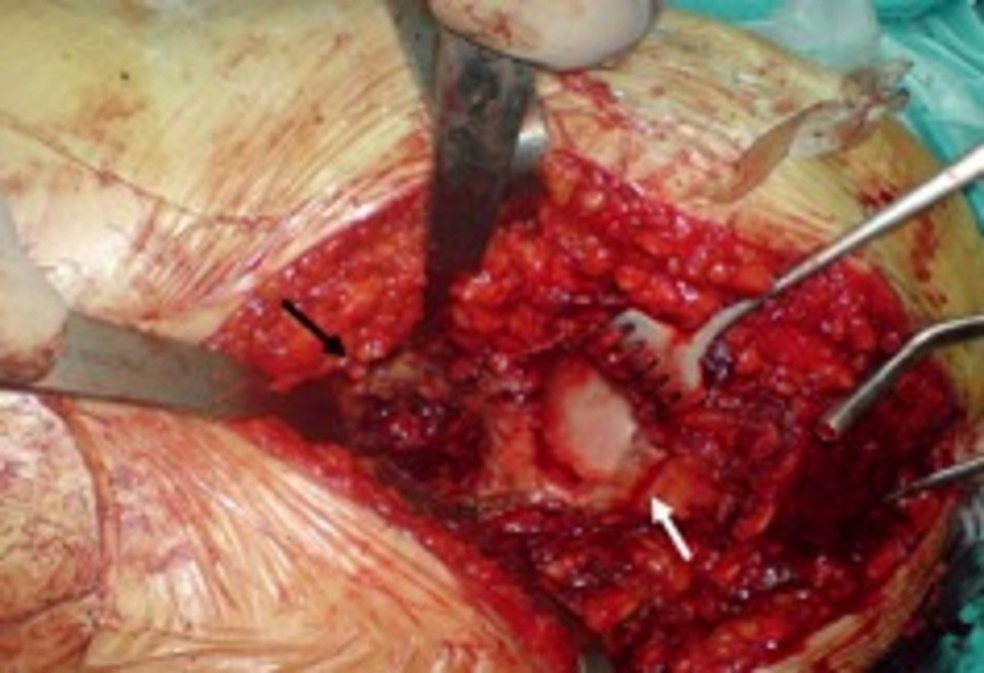
Intraoperative image of a patient with high dislocation. The false acetabulum is indicated with the white arrow. The native, hypoplastic acetabulum is indicated with the black arrow.

**Figure 3:**
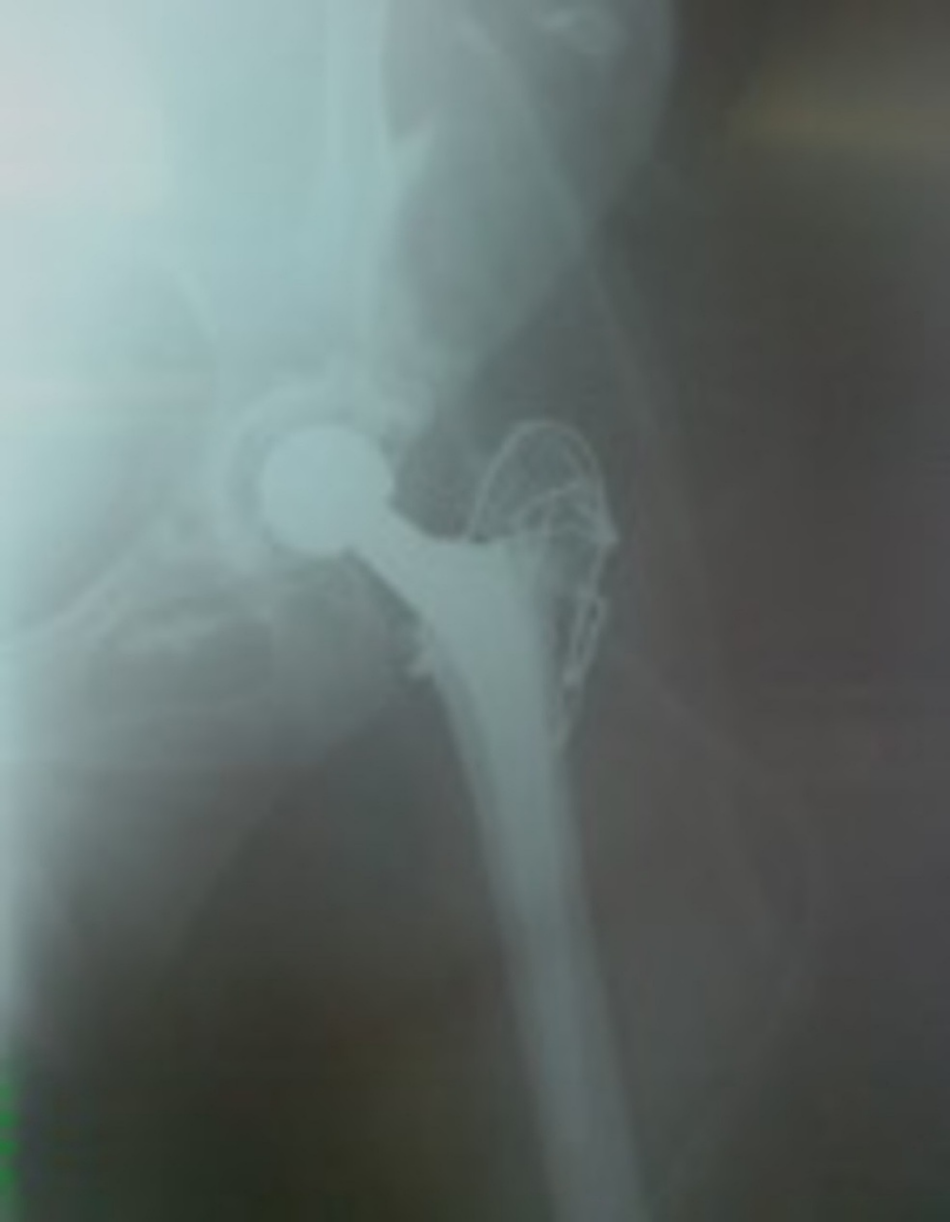
Postoperative image of the same patient. A trochanteric osteotomy was performed in order to facilitate the positioning of the hip joint to the lower native place.

**Figure 4:**
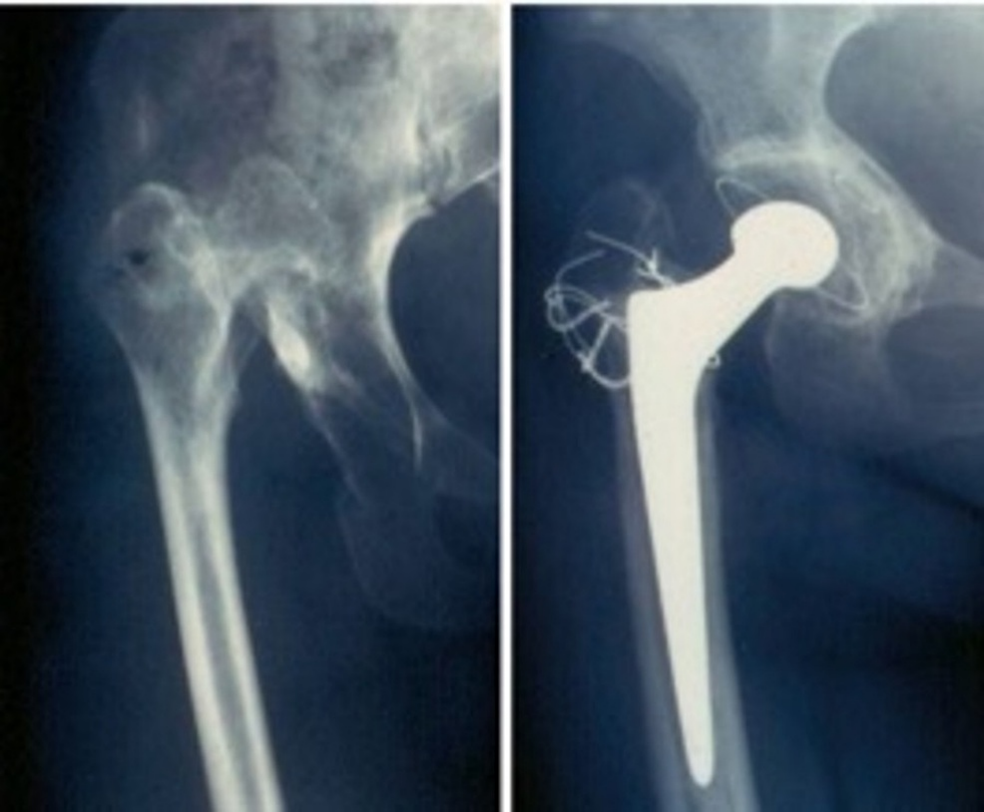
High CDH, before and after arthroplasty. Cotyloplasty was necessary for the accommodation of the artificial cup.  Nine years follow-up. Note the remodelling of the acetabulum roof.

Aseptic loosening is considered one of the leading causes for revision, especially in the younger populations due to the increased activity levels [34]. Li et al. [27] reported that the acetabular revision rates for aseptic loosening were 8% at five years and 26% at 10 years. Inao and Matsuno [28] reported aseptic loosening in three sockets (15%) but not in the stems. The cumulative survival rate at 20 years was 78%, with revision for loosening of the acetabular component as the end-point. However, new technologies regarding personalised and customized implants have also been used for DDH patients, as a specific guide using acetabular superolateral rim during THA for DDH is used to assist the production of an artificial acetabulum in adult DDH patients [37] . Wang et al studied the patient-specific instrument (PSI) in a study of 20 THA patients with DDH, leading to satisfying radiological and functional post-operative results (Figures 2, 3, 4) [43]. Additionally, the use of Metha short hip stem has drawn attention, as it is likely to bridge the gap between traditional straight design stems and hip resurfacing prostheses in THA in young DDH patients. The Metha short hip stem system is an uncemented neck-retaining monoblock or modular stem, which is made of a titanium alloy with a proximal rough titanium surface, with a proximal trapezoidal section allowing multipoint contact and providing 3-point fixation through the medial calcar region, the proximal lateral cortex and proximal posterior cortex [44,45].

## Conclusions

Through a respectable number of publications over the last two decades, it seems that the challenges in DDH can be satisfyingly addressed using specifically designed implants, bone grafts and appropriate osteotomies. Placement of the cup in its anatomical location is a key factor, as it allows for restoration of the abductor muscles lever arm and normal hip center, thus providing superior outcomes in terms of biomechanics. Leg lengthening and equalization are crucial elements of the reconstruction. Historically, the outcomes of THA in DDH were reported to be poorer than those in hips without DDH. Nevertheless, current evidence suggests a trend of continuously improving outcomes in terms of pain relief, functional improvement, return to daily activities and reduction in complications rate. Unfortunately, there is a lack of high-quality studies to establish the optimal acetabular and femoral components. Future research should also focus on the impact of THA on the quality of life of this particular population.
